# Simultaneous Bilateral Patellar Tendon Rupture in a Patient With Osteogenesis Imperfecta

**DOI:** 10.7759/cureus.15466

**Published:** 2021-06-05

**Authors:** Matthew H Nasra, Christopher Dijanic, Suleiman Sudah, Christopher R Michel, Jason Cohen

**Affiliations:** 1 Orthopaedic Surgery, Rutgers Robert Wood Johnson Medical School, New Brunswick, USA; 2 Orthopaedic Surgery, Monmouth Medical Center, Long Branch, USA

**Keywords:** knee, simultaneous, osteogenesis imperfecta, patellar tendon, rupture, bilateral

## Abstract

Simultaneous bilateral patellar tendon rupture is an infrequent and debilitating injury. Although tendon rupture is associated with multiple systemic diseases, there is limited literature regarding its association with osteogenesis imperfecta and successful treatment techniques. We report a case of a 56-year-old man with a history of osteogenesis imperfecta type I who experienced bilateral patellar tendon rupture following a fall from standing height in the absence of other risk factors. Both injuries were effectively treated with primary open repair utilizing Krackow suture technique and transosseous tunnel fixation bilaterally. The patient demonstrated full functional recovery at 14-month follow-up. Osteogenesis imperfecta is a risk factor for the development of bilateral patellar tendon rupture. Open bilateral transosseous suture repair has proven to be successful despite defects in type 1 collagen and underlying tendon weakness.

## Introduction

Simultaneous bilateral patellar tendon rupture is a devastating and rare injury, disrupting the knee's extensor mechanism [1-3]. While this event is most often a result of major trauma, injury in the setting of minimal trauma or spontaneous rupture is even more uncommon [1,3]. Tendon rupture can occur in the presence of risk factors such as systemic disease, oral corticosteroid use, and steroid injections [4]. Osteogenesis imperfecta (OI) is a genetic disorder characterized by defective collagen [5]. Although tendon and ligamentous injury is known to occur in this population [6,7], few cases of simultaneous bilateral patellar tendon rupture associated with OI exist in the English literature [8-10]. Here, we report a case in a 56-year-old male with a history of OI and the absence of other recognized risk factors. He was successfully treated with open bilateral transosseous repair two days following the injury. Despite the extreme rarity of these events, the association between OI and simultaneous bilateral patellar tendon rupture should be further explored, given the severely debilitating nature of this injury.

## Case presentation

A 56-year-old man with a history of OI type I, hearing impairment, and blue sclera presented with bilateral knee pain and an inability to ambulate. He experienced a fall while showering, causing him to land directly onto both patellae. The patient denied any other medical history.

**Figure 1:**
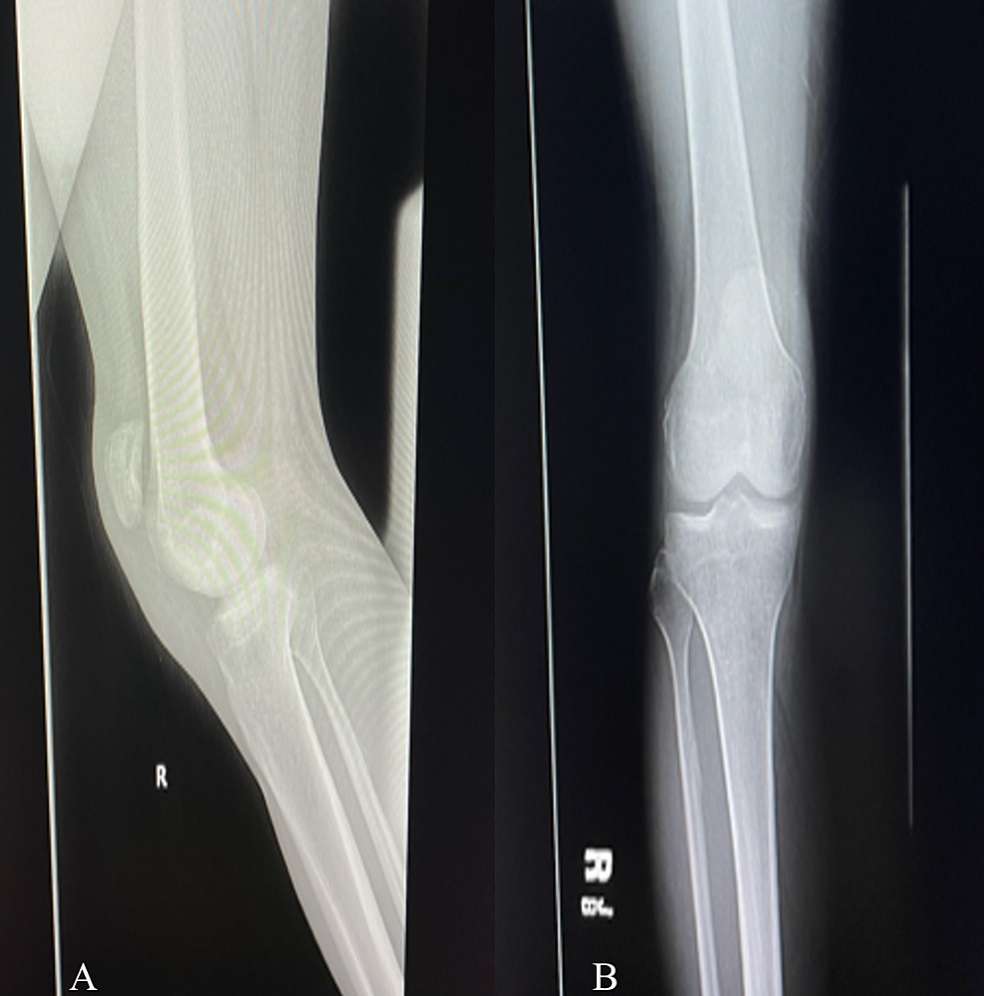
Radiograph of the right knee: (A) lateral view and (B) anteroposterior view showing the high-riding patella.

**Figure 2:**
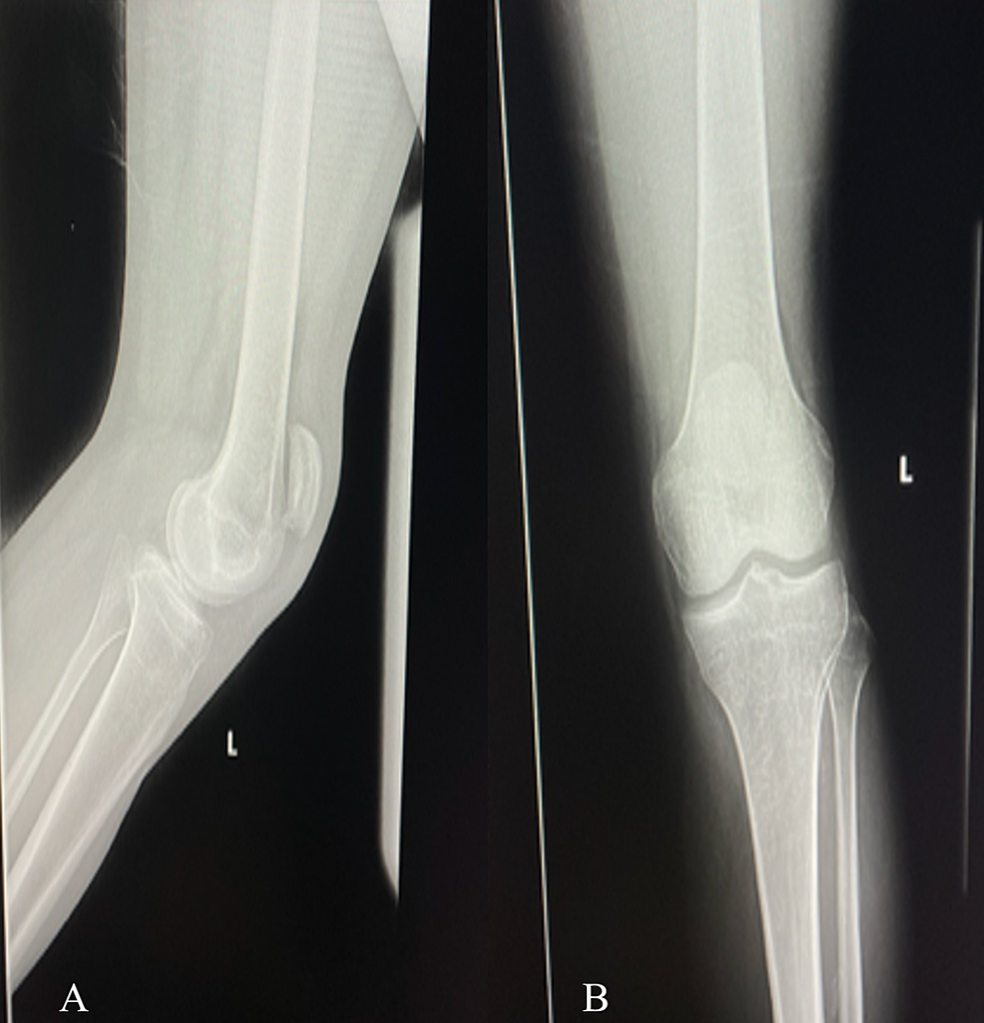
Radiograph of the left knee: (A) lateral and (B) anteroposterior view showing the high-riding patella.

Upon physical examination, both patellae were subluxated superiorly, and mild effusions were present. The patellar tendons were non-palpable bilaterally. No step-offs were appreciated on the anterior surface of the patellae. He had a full passive range of motion (ROM) of both knees but was unable to actively straight leg raise bilaterally. Plain radiographs revealed diffuse osteopenia and bilateral patella alta; no gross fracture or obvious avulsion was identified (Figures 1A, 1B, 2A, 2B).

**Figure 3:**
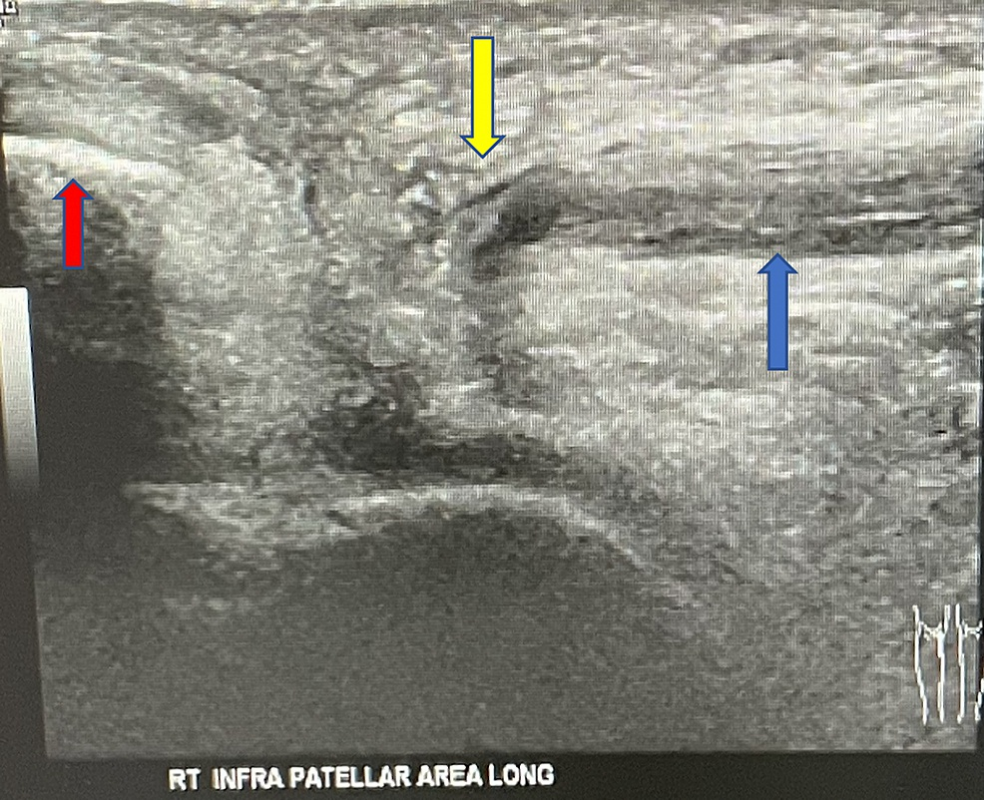
Ultrasound of the right knee showing patellar tendon rupture. Non-visualization of the right patellar tendon (blue arrow) to its attachment at the inferior pole of the patella (red arrow). The area of tendon disruption is indicated by the yellow arrow.

**Figure 4:**
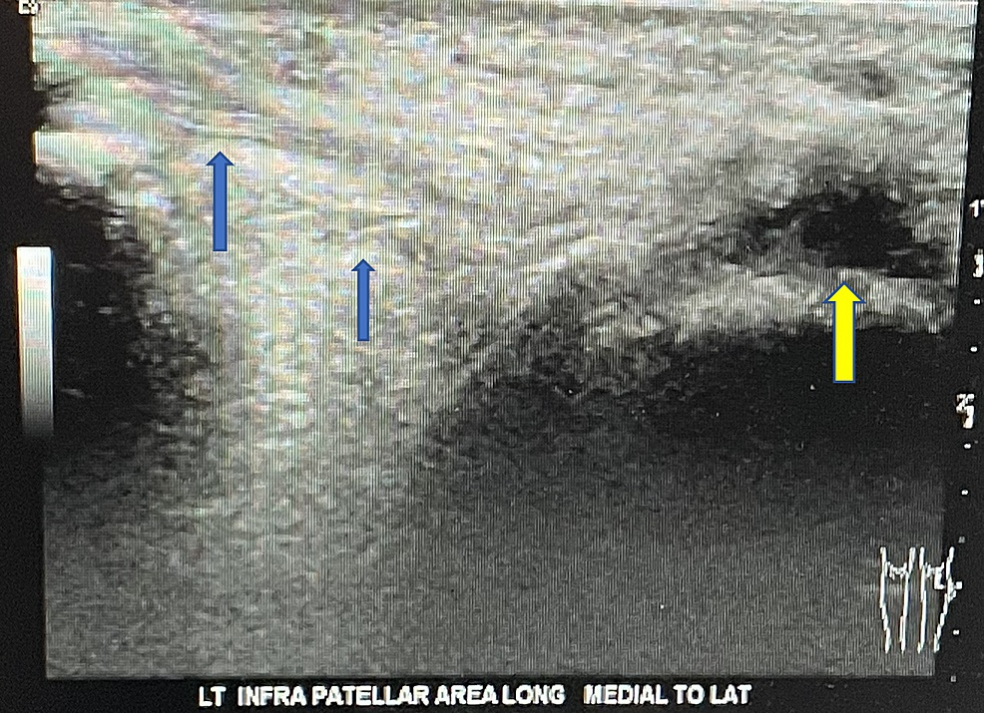
Ultrasound of the left knee showing patellar tendon rupture. No normal-appearing patellar tendon is visualized within its expected location. The attachment at the inferior pole of the patella appears attenuated, with fibers seen extending anterior to the patella (blue arrows). The tibial tuberosity is indicated by the yellow arrow.

Sonography of the bilateral lower extremities confirmed the diagnosis of bilateral patellar tendon rupture (Figures 3, 4).

He was later treated with an open primary repair of both tendons.

Surgical technique

The patient was placed in the supine position and draped in the typical sterile fashion. Two grams of cefazolin were administered preoperatively, and tourniquets were placed on both legs, neither of which was utilized. A midline incision with full-thickness skin flaps was performed extending from the superior pole of the patella to the tibial tubercle beginning with the left lower extremity. Complete disruption of the retinaculum and patellar ligament at the inferior pole of the patella was identified. A tear was present along the proximal aspect of the tendon, which formed a proximal stump in this area. The edges of the distal tendon and inferior pole of the patella were sharply debrided, and transosseous tunnels were drilled through the patella. Two #5 (7.0 metric) Ethibond (Ethicon Inc., Somerville, NJ, USA) sutures were placed along the length of the distal tendon using a running locking Krackow technique, pulled through the transosseous patella tunnels from an inferior to superior direction, and secured in traditional fashion. The proximal stump created by the tear of the proximal tendon was carried pants-over-vest style anterior to the primary repair using a Krackow stitch and secured to either side of the tibial tubercle with two Swivel-lock anchors. The knee was then ranged through 90 degrees of flexion without significant gapping. The surgical site was closed using #1 Vicryl (Ethicon Inc.) sutures for the retinaculum repair, followed by #0 Vicryl (Ethicon Inc.) sutures, 2-0 Vicryl (Ethicon Inc.) sutures, and sterile skin staples. Attention was then turned to the right lower extremity, with the same surgical exposure utilized. Patellar tendon avulsion from the inferior pole was the only identified injury. The repair of the right side proceeded identically to the left, except for addressing a proximal stump.

Postoperative course

The patient was discharged with bilateral hinged knee braces postoperatively and instructed to conduct active ROM exercises from zero degrees to 30 degrees for the first six weeks, zero degrees to 90 degrees for the next six weeks, and zero degrees to 120 degrees thereafter. Ambulation was to be performed with the knees locked in full extension during the first six weeks, with advancements of 30 degrees of flexion every six weeks throughout the recovery period.

By two months, the patient was able to ambulate independently without the use of an assistive device. Full extension and active flexion to 85 degrees were achieved bilaterally without subluxation of the patellae. Quadriceps strength was grade 4, Medical Research Council (MRC) Scale.

By six months, the patient experienced notable improvement. The pain-free joint motion was maintained, active flexion was 110 degrees bilaterally, and quadriceps strength was 4/5.

At the 14-month follow-up visit, the patient had a full ROM of the knee joint with a return to pre-injury activity level. He denied any residual pain symptoms or other complications at the time of follow-up.

## Discussion

Patellar tendon rupture is a debilitating injury that causes significant dysfunction of the entire extensor mechanism of the knee [2]. These injuries are commonly associated with athletic activity or trauma and occur following eccentric overload with the knee in a flexed position [11,12].

Bilateral patellar tendon rupture is an infrequent event. A recent analysis presented a total of 48 cases, 16 of which occurred in the absence of trauma or athletic exertion [3]. Risk factors included oral or injectable corticosteroid use and systemic diseases, such as lupus, hyperparathyroidism, chronic kidney disease, rheumatoid arthritis, gout, diabetes, and obesity [3,4,11]. Our patient denied a history of any of these risk factors. It has been proposed that conditions that weaken collagen can predispose patients to tendon rupture [1,3,4,8-12].

OI is a genetic connective tissue disorder characterized by defects in type 1 collagen synthesis. The disease manifests clinically in tissues that have type 1 collagen as the principal matrix protein (bone, dentin, sclerae, tendon, and ligaments) [5,10]. This disorder has been associated with bone fragility, ligamentous laxity, tendon rupture, and underlying tissue weakness as each of these structures is mainly comprised of type 1 collagen [5-10]. Diffuse osteopenia associated with multiple fractures and deformities is a radiographic hallmark of OI, with almost all cases having some form of generalized osteopenia [5]. The diagnosis of OI is made with clinical and radiographic evidence. Type I OI is the most common form and displays an autosomal dominant inheritance pattern [5]. Patients typically experience multiple, recurrent fractures from an early age. Tendon ruptures occur in this population although less frequently [7].

Patellar tendon rupture in patients with OI is extremely rare. Most cases are unilateral and occur in the context of athletic activity or trauma [6,7,11]. Few cases of OI-associated simultaneous bilateral patellar tendon rupture exist in the English literature [8-10]. Kothari et al. [8] reported a case in a 27-year-old woman with OI type I who sustained the injury after a trip and fall onto both knees while walking. The authors attributed the injury to diminished tendon strength as a result of the patient’s OI. This injury mechanism was similar to our patient, who also landed on both knees following a ground-level fall. Additionally, Kim et al. [9] reported a case in a 55-year-old woman with OI type I that occurred while working without obvious trauma. The authors attributed this injury to the brittle bone, collagen matrix abnormalities, and tendon weakness from her underlying OI [9]. Given this history of minimal trauma, these patients likely experienced a considerable degree of tendon weakening. In the absence of other established risk factors, we believe that the metabolic and structural collagen defects imposed by OI served as causative factors in the development of simultaneous bilateral patellar tendon rupture in our patient.

Previous cases of patellar tendon rupture in patients with OI have been managed successfully with both traditional and augmented repair techniques. Ogilvie-Harris and Khazim [7] reported two cases of unilateral patellar tendon rupture in patients with OI type I treated with a standard repair using suture anchors. Abnormalities in type 1 collagen resulted in diminished tendon strength and subsequent rupture. Both patients experienced functional improvement: one patient had full ROM at six months and the other a full return to sports at two years [7].

Jansen and Haddad [11] described a case of a distal patellar tendon avulsion fracture in a patient with OI type I. The repair was done using Ethibond (Ethicon Inc.) sutures looped through tibial tubercle drill tunnels. Augmentation with FiberWire (Arthrex Inc., Munich, Germany) was used to protect the repair and allow for early mobilization in view of the patient’s OI [11]. At the one-year follow-up visit, the patient was pain-free and achieved 120 degrees of flexion and terminal extension. Similarly, Kothari et al. [8] and Kim et al. [9] both described a case of successful bilateral patellar tendon repair using Ethibond (Ethicon Inc.) sutures and a wire loop for reinforcement with good postoperative outcomes. One patient experienced symptom-free full ROM bilaterally at seven months [8]. The other patient was able to return to a similar activity level comparable to her baseline prior to the injury [9].

Despite these results, long-term repair integrity in this population raises appropriate concern. Elguindy et al. [6] reported a case of patellar tendon rupture treated with primary tendon repair and augmentation with a turndown of the quadriceps tendon. This procedure was unsuccessful and required revision with allograft reconstruction at 18 months due to failed restoration of the extensor mechanism. At the final follow-up visit, the patient experienced a full recovery with no activity restriction and a return to his pre-injury level of activity [6]. The tendon rupture, in this case, was attributed to progressive collagen deficiency as a result of OI. This case suggests that primary repair may be successful up to a specific point within this patient population, after which allograft reconstruction may become necessary.

## Conclusions

Simultaneous bilateral patellar tendon rupture is an infrequent and debilitating injury, with limited literature on the association with OI. Our case supports OI as a direct risk factor for simultaneous bilateral patellar tendon rupture in the presence of minimal trauma. Moreover, we find that utilizing open repair with the Krackow suture technique and transosseous tunnel fixation is an effective method for acute treatment in this population. Future studies must be conducted to evaluate the extent of tendon compromise as a factor of disease progression, as long-term data related to repair integrity is lacking, and questions regarding optimal surgical management - primary repair, primary repair with augmentation, or primary reconstruction - remains.
